# Kala-Azar: A Case Report

**DOI:** 10.7759/cureus.34864

**Published:** 2023-02-11

**Authors:** Nina Jancar, Filipa Sousa Gonçalves, José Duro, Inês Pinto, Tiago Oliveira, Patrício Aguiar

**Affiliations:** 1 Internal Medicine, Hospital de Santa Maria, Centro Hospitalar Universitário Lisboa Norte, Lisbon, PRT; 2 Pathology, Hospital de Santa Maria, Centro Hospitalar Universitário Lisboa Norte, Lisbon, PRT; 3 Medicine Department, Faculty of Medicine, Lisbon University, Lisbon, PRT

**Keywords:** unintentional weight loss, immunocompetent adult, high fever, “pancytopenia”, visceral leishmaniasis (vl)

## Abstract

Leishmaniasis is a zoonosis caused by unicellular protozoans *Leishmania*. The transmission can be zoonotic or anthroponotic, depending on the species, and the main vector is the phlebotomine sandfly. The disease is endemic in the tropics of Asia and Africa but is considered rare in Portugal, especially in immunocompetent hosts. Its main clinical syndromes constitute cutaneous leishmaniasis, mucocutaneous disease, and visceral leishmaniasis. The latter is also known as *kala-azar* and is caused by the infection of the phagocytes of the reticuloendothelial system, causing the typical symptoms: fever, hepatosplenomegaly, and pancytopenia. The clinical manifestations are non-specific, frequently causing a delay in the diagnosis, especially in nonendemic areas and immunocompetent hosts. Early diagnosis and treatment are essential, given the high mortality rate in untreated patients. The diagnosis is based on the direct visualization of the protozoan and molecular methods, such as polymerase chain reaction tests. Amphotericin B is considered the first-line treatment. We present a case of visceral leishmaniasis in an immunocompetent patient with fever, hepatosplenomegaly, and pancytopenia.

## Introduction

Visceral leishmaniasis, also known as *kala-azar,* is a systemic disease caused by unicellular protozoans of the genus Leishmania (principally *Leishmania donovani* (*L. donovani)* and *Leishmania* *infantum (L. infantum)*, depending on the geographical area), transmitted by phlebotomine sandflies [1-3]. The disease is endemic in the rural areas of the tropics of Asia, East Africa, and Brazil [3] but is hypoendemic in Portugal [4]. The principal clinical syndromes associated with the Leishmania infection are cutaneous leishmaniasis, mucocutaneous leishmaniasis, and visceral leishmaniosis, caused by systemic dissemination of the parasites through the reticuloendothelial system [1-3]. Clinical manifestations of visceral leishmaniasis are non-specific [3], and a high index of suspicion is necessary to diagnose the disease in non-endemic areas. When untreated, the disease is associated with a high mortality rate [2].

## Case presentation

We present a case of a 38-year-old woman with a history of primary hyperparathyroidism, having undergone parathyroidectomy and depression, for which she was medicated with escitalopram and bromazepam. The patient presented with vespertine fever, night sweats, weight loss (6.1% of the total body weight), anorexia, and non-productive cough, which started about a month before the hospitalization. The patient was of African descent and had moved to Portugal from the Democratic Republic of São Tomé and Príncipe (not an endemic area for leishmaniasis) three years before the hospitalization and had traveled there six months before the onset of symptoms. There was no other relevant epidemiologic history, such as insect bites, contact with dogs, or other domesticated or wild animals. 

At admission, pallor of the mucosa and splenomegaly were identified. Laboratory investigations revealed new-onset pancytopenia (normocytic anemia 10.3g/dL, leukopenia 2700/μL, and thrombocytopenia 90000/μL) and C-reactive protein elevation (14mg/dL) (Table 1).

**Table 1:** Laboratory tests performed at admission, showed pancytopenia CRP: C-reactive protein; IGRA: Interferon-gamma release assay

Laboratory test	Value at admission	Normal range
Hemoglobin	10.3g/dL	12-15.3g/dL
Leukocytes	2700x10^9^	4000-11000x10^9^
Platelets	90000 x10^9^	150-450 x109
CRP	14mg/dL	<0.5 mg/dL
IGRA	Negative	-

The peripheral blood smear was unremarkable, and blood infectious serologies, including human immunodeficiency virus (HIV), hepatitis B and C, cytomegalovirus (CMV), parvovirus B19, Epstein-Barr virus (EBV), as well as *Brucella*, *Coxiella burnetii*, *Rickettsia conorii* and the enzyme-linked immunoassay test for *Leishmania*, were all negative (Table 2). Malaria parasites were not found on the peripheral blood smear. The Interferon-γ release assays (IGRA) test was negative as well.

**Table 2:** Infectious serologies solicited

Infectious serologies	Result
Human immunodeficiency virus (HIV)	Negative
Hepatitis B	Negative
Hepatitis C	Negative
Parvovirus B-19	Negative
Cytomegalovirus (CMV)	Negative
Epstein-Barr virus (EBV)	Negative
Brucella spp.	Negative
Rickettsia conorii	Negative
Coxiella burnetii	Negative
Leishmania spp.	Negative

**Figure 1:**
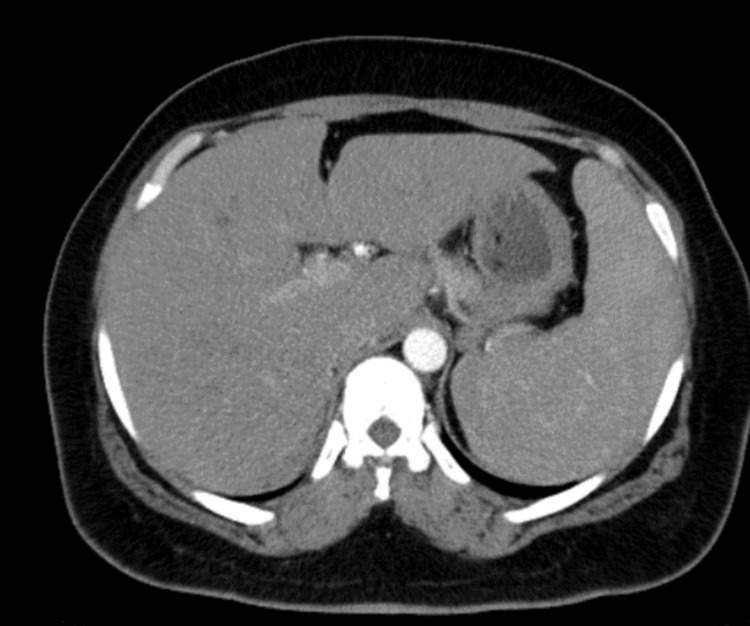
Abdominal CT scan showing hepatosplenomegaly CT: computerized tomography

The whole-body computerized tomography (CT) showed discrete hepatosplenomegaly and para-aortic lymphadenopathy (Figure 1). Given the persistent fever and pancytopenia during the hospitalization, a positron emission tomography (PET) was solicited, and intense metabolic activity of the spleen and bone marrow was detected.

**Figure 2:**
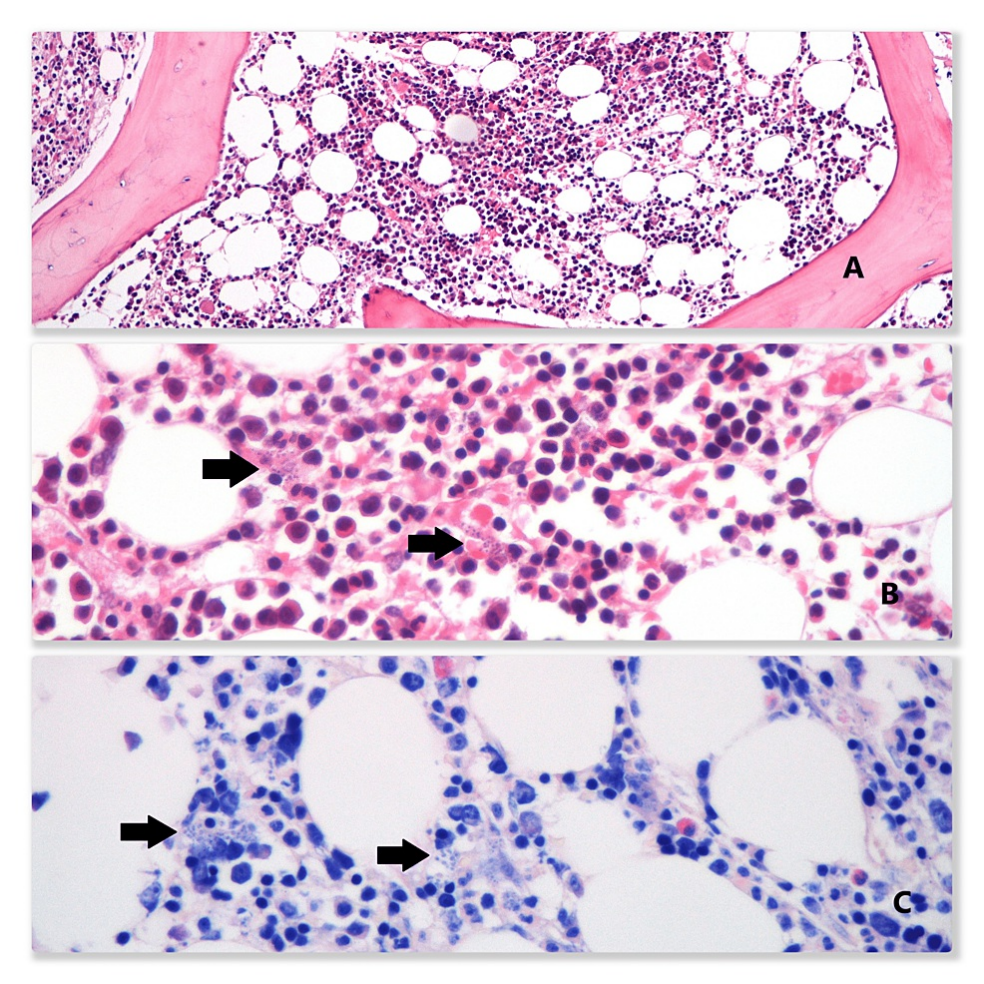
Microscopical analysis of the bone marrow that shows normal cellularity (A). At 40x magnification, numerous macrophages with round and basophilic intracytoplasmic organisms that corresponded to Leishmania amastigotes are seen (B). These organisms are also readily visualized in a Giemsa staining (C)

A myelogram and bone marrow biopsy were performed, evidencing multiple Leishmania, thus confirming the diagnosis of visceral leishmaniasis or kala-azar (Figure 2).

Treatment with intravenous liposomal amphotericin B (3mg/kg/day, for five days) was started, with the resolution of the fever, thrombocytopenia, and leucopenia, maintaining only anemia (10g/dL) on hospital discharge.

## Discussion

Visceral leishmaniasis (VL) is a systemic disease caused by unicellular protozoans from the Leishmania genus, typically transmitted by female phlebotomine sandflies [1]. Although nearly 90% of all worldwide cases occur in Bangladesh, India, Nepal, Sudan, and Brazil, Leishmania is endemic in some Mediterranean and Middle East countries [5]. VL is typically caused by *L*. *donovani*, found almost exclusively in the Old World, and *L*. *infantum*, endemic in Latin America and the Mediterranean [6]. The transmission is either anthroponotic, in the case of *L*. *donovani*, or zoonotic, as in *L*. *infantum*, with dogs, rodents, foxes, and other animals serving as the main reservoir [2,6]. Despite the geographic localization, visceral leishmaniosis is hypoendemic in Portugal, with an incidence rate of 0.1 cases per 100,000 inhabitants [4], and is more frequently diagnosed in the pediatric population and immunocompromised patients [7].

The incubation period can vary between 2 weeks and 18 months, but the disease can take years to become symptomatic [6]. After the inoculation of the promastigote form into the skin, the parasites are internalized by phagocytes (dendritic cells and macrophages) in the skin, where they transform into amastigotes, replicate, and later disseminate through lymphatic and blood vessels and infect the phagocytes of the reticuloendothelial system [2-3], resulting in infiltration of the bone marrow and hepatosplenomegaly [3]. The most common clinical manifestations are fever, weight loss, fatigue, and hepatosplenomegaly [8], but the symptoms may be atypical in immunocompromised (especially AIDS-acquired immunodeficiency syndrome) patients [2]. As the disease progresses, fatigue aggravation due to anemia and abdominal pain due to hepatosplenomegaly may occur [2-3,8]; gastrointestinal involvement, causing diarrhea, pleural effusion, and pulmonary nodules, are also described [5].

Typical laboratory findings include anemia, leukopenia, pancytopenia, increased transaminases, and hypergammaglobulinemia [5,8]. A high index of suspicion is necessary to diagnose VL since its clinical presentation is non-specific and can be associated with other infectious diseases, such as tuberculosis, malaria, and brucellosis, as well as hematologic malignancies and macrophage activation syndrome [5,8].

Traditionally, the gold standard for the diagnosis has been direct visualization of the parasite on microscopy or culture of invasive samples [9,10]. Still, molecular methods have the highest sensitivity and specificity [10]. Therefore, a step-wise approach is recommended in the diagnosis of VL [9]; non-invasive tests, such as serology and polymerase chain reaction (PCR) tests, should be the first to be performed [9]. In contrast to immunocompromised patients, serology has good sensitivity and specificity (80%-100%) in immunocompetent patients [5,9]. When the diagnosis can’t be ruled out or reliably confirmed, bone marrow, lymph nodes, or spleen samples should be obtained [9]. Spleen biopsy has the best sensitivity (93%-99%) [9], but because of the risk of spleen hemorrhage, bone marrow aspirate is preferred, despite the lower sensitivity (53%-86%) [5,9]. RK39 and rK28-based rapid diagnostic tests have been implemented, but their sensitivity and specificity vary according to the geographical location, being highest in the Indian continent [9-10] and, thus, not very useful to diagnose VL in Portugal.

Our patient was of African descent and had moved to Portugal from the Democratic Republic of São Tomé and Príncipe three years before the hospitalization; she had visited the country about six months before the hospitalization. According to the World Health Organization (WHO), no autochthonous cases have been reported in the country up until now [11]. No insect bites or contact with dogs or other domesticated or wild animals were reported. She presented with typical symptoms of intermittent fever, weight loss (6.1%), night sweats and fatigue, and splenomegaly was observed at admission. The laboratory results confirmed pancytopenia. Although she presented a typical clinical picture, it is not specific to leishmaniasis, and the patient did not refer high-risk epidemiologic history for leishmaniasis.

Other parasitic and bacterial infections were excluded, including malaria, an endemic disease in São Tome, tuberculosis, brucellosis, and rickettsiosis. Viral infections, causing fever and pancytopenia, were also ruled out. The serology for Leishmania in the peripheral blood was negative. Further investigation was performed to confirm leishmaniosis and exclude eventual hematologic disease. Hepatosplenomegaly was documented on the CT scan; intense metabolic activity of the spleen and bone marrow was detected on the PET scan. Given the high risk of post-procedure hemorrhage, a bone marrow biopsy and myelogram were performed instead of a spleen biopsy. Multiple Leishmania parasites were observed on microscopy, confirming the diagnosis of VL. 

When not treated, VL is associated with a high mortality rate [10]. Therefore, all symptomatic patients should be treated according to the current guidelines, and the treatment should be adapted to the patient’s immune status and the geographic location where the infection was acquired [1,9]. Currently, amphotericin B and miltefosine (for *L. donovani*) are considered the first-line treatment, with pentavalent antimony being an alternative treatment in patients that do not tolerate the treatments mentioned above [1,9]. Clinical parameters correlate with the response to the treatment, and invasive methods are not recommended to monitor the response [1]. Our patient was started on liposomal amphotericin B (3mg/kg/day intravenously for five days, and on days 14 and 21) after an infectious disease consult, with the resolution of fever and normalization of thrombocytopenia and leukopenia, maintaining discrete anemia. 

## Conclusions

Visceral leishmaniasis is a systemic, potentially fatal disease caused by protozoa of the *Leishmania genus*. It is transmitted by sandflies, and its natural reservoir is dogs, foxes, and rodents. Due to its non-specific clinical presentation, a high index of suspicion is necessary to diagnose the disease, especially in non-endemic areas and immunocompetent patients. A prompt diagnosis and exclusion of other infectious and hematologic diseases are crucial. The timely institution of treatment has a direct impact on the prognosis. Direct visualization and molecular tests are the most sensitive and specific tools for diagnosis.
